# Global Emergency System Based on WPAN and LPWAN Hybrid Networks

**DOI:** 10.3390/s22207921

**Published:** 2022-10-18

**Authors:** Eduardo Pérez, Raúl Parada, Carlos Monzo

**Affiliations:** 1 Faculty of Computer Science, Multimedia and Telecommunications, Universitat Oberta de Catalunya (UOC), Rambla del Poblenou 156, 08018 Barcelona, Spain; 2 Centre Tecnològic de Telecomunicacions de Catalunya (CTTC), Av. Carl Friedrich Gauss, 7-Edifici B4, 08860 Castelldefels, Spain

**Keywords:** IoT, WPAN, LPWAN, emergency, fire detection, heterogeneous network

## Abstract

There are multiple methods of communication for the transmission of every type of alarm. In the case of emergency systems, they are usually controlled by private companies that work as bridges between the source and the receiver of an emergency. Furthermore, it is necessary to use an independent communication system for each building, requiring human vigilance, leading to an increase in infrastructure and service costs as well as response time. This paper proposes a hybrid network by combining both wireless personal access network (WPAN) and low power wide access network (LPWAN) communication for the complete development of a communication architecture oriented to an emergency system. The main aim of this work is to provide a global emergency system that is focused on fire detection but is also suitable for other critical events, with low energy consumption, a wide communication range covering up to 50 km, and a low cost of service and infrastructure. This proposal reduces the total energy consumption of the system with respect to typical fire detection systems.

## 1. Introduction

Any home or property, as well as the people who live there, are subject to different dangers caused by external factors. These threats vary from infrastructure failures to human errors, all of them endangering the lives of the inhabitants or causing material damage. This amount of damage will be proportional to the response time of the emergency services [1]. Therefore, early detection is key to minimizing material and human damage.

**Figure 1:**
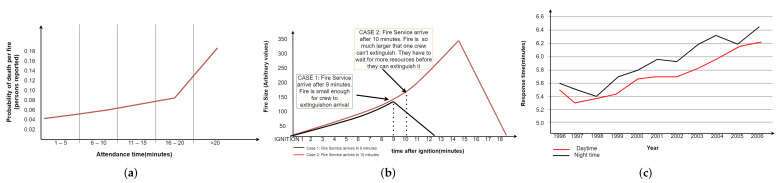
Revealing historical data of the evolution in fire detection and the relevance of response time with survival. (**a**) Relationship between fire death and the exposure time of fire appliances; (**b**) effect of a small increased exposure time; (**c**) day and night time dwelling fire response times [1].

According to [1], Figure 1 shows the real importance of the response time of the emergency services in a case of fire. Figure 1a shows that the probability of death from a fire increases when the response time of the emergency services increases. Figure 1b shows the consequences in fire size when the response time is increased and how this increment is not linearly proportional. Figure 1c shows a global growth in response time measurement year by year, both day and night. With current detection systems relayed to someone notifying emergency centers, if there are humans involved, around 80% of the cases are fatal, even before a warning is sent.

In recent years, new wireless communication technologies have been incorporated into communications, especially focused on the Internet of Things (IoT) field. These technologies have shown great growth in terms of investment, with a projection of 1463 billion dollars in 2027 [2]. The growth of devices of this type is estimated to reach 75 billion connected devices by 2025 [3]. The combination of these communication technologies can allow the development of open communication architectures with great improvements over conventional architectures. In the following sections, we show one application with the latest wireless communication technologies with distinctive characteristics such as wireless personal area networks (WPANs) and low-power wide area networks (LPWANs), allowing for the design of an emergency system aimed to improve the relevant parameters in communication systems, such as robustness, coverage, economic cost, energy consumption, and response time. Therefore, this work aims to take advantage of the technological progress for the development of a hybrid network that addresses all the previous points of interest for the improvement of a global emergency system.

Currently, there are solutions on the market oriented to emergency detection systems. These systems are based on communication technologies that are characterized by high power consumption [4,5,6,7], depend on the user’s own infrastructure, and entail an excessive cost for the service offered by the supplier company, which owns the technology used.

New technologies create the opportunity to improve global detection systems by making them more accessible and easier to set up. Therefore, we focused on the development of an open, flexible, and accessible system. We consider the following requirements: (i) the use of common and open communication technologies and (ii) the development of electronic equipment at a low cost and with great autonomy as well as the reuse of infrastructures already established to reduce costs.

This paper is organized as follows. Section 2 reviews the state of the art of current solutions in the field of emergency systems. Section 3 describes the proposal of this paper: an emergency system based on a hybrid wireless communication system, the architecture, and its most relevant features. Section 4 presents the main advantages of the proposed system over current emergency systems and includes experimental results. Finally, the paper results are summarized and conclusions with potential future work are detailed in Section 5.

## 2. State of the Art

This section presents the most advanced references in terms of security-oriented communications related to the work conducted. Ferreira et al. [8] proposed a low-power system. Their proposal was a wireless communication architecture made up of two technologies: Bluetooth as WPAN technology creating the network between sensors, and LoRa (long range) as LPWAN technology routing the data to the cloud. Bluetooth low energy (BLE) technology allows great connectivity between nearby devices at a low energy cost, while long-range wide area network (LoRaWAN) technology allows wide coverage communications. Its maximum communication range with the system connected to the cloud is 15 km, while the nodes have a maximum coverage of tens of meters. The global electric consumption of the system is low; this type of technology aims to optimize its energy efficiency, although the gateways need to be permanently powered to be able to continuously listen to the requests of the nodes. Its greatest disadvantage is the initial cost of implementing the infrastructure, since it is necessary to install the entire communication system and repeat the signals.

A second related work is a system designed by the company Semtech [9], which owns the LoRa transceiver technology. It proposes a wireless communication system made up exclusively of devices with LoRa connectivity. The sensors are connected in a star topology to a gateway that will grant access to a server in the cloud. This system focuses on reducing energy consumption as much as possible, since the node, providing its own LoRa transceiver, allows for optimal control of communication and idle states. Regarding its coverage, this architecture has great coverage even though it is a single-level system. On the contrary, the economic cost is much higher than other solutions due to the cost of each device. A LoRa transceiver is several times more expensive than a Bluetooth module, which is commonly already incorporated in the processing unit.

Muheden et al. [10] proposed a detection system with wireless local area network (WLAN) connectivity. In particular, a wireless LAN 802.11 b/g can be used. This is an already conventional technology that exists in any home. Their proposed approach focuses on the implementation of sensor devices with Wi-Fi connectivity as a way of communicating alarms to the user through the mobile phone. Regarding the architecture of the network, in this case, it is mainly based on a home network in which the sensor devices are distributed throughout the home and connected through a gateway, the home router, which will provide external connectivity to the Internet, or connectivity to any client device in the home, such as a computer or mobile phone that can receive the notification. This system stands out for its low initial infrastructure cost, although there is a fixed cost of service for the use of an Internet provider for the communication of the system with the cloud. In addition, it is a system with high energy consumption, so the devices have reduced portability, as they must be plugged into to the power grid.

There are numerous companies that offer a comprehensive security service, for instance, ADT [11], Sector Alarm [12], and Prosegur [13]. One of the optionally included services is a fire detection module based on gas or temperature sensors. An example of this system is presented by Heyitech Alarms [14] and is based on a WLAN communication system, forming an internal local network between the different devices connected to the center from a proprietary technology based on a WPAN, such as Zigbee and Bluetooth, or a WLAN, such as Wi-Fi, such that all information captured from the different control points of the house can be transmitted. The control center transmits all information captured by the sensors to the surveillance center, where a user decides to communicate with relevant emergency authorities based on the acquired sensor data. The information transmission method between the home office and the emergency unit will be carried out using mobile technology, GSM, GPRS, or LTE, giving the possibility of sending enormous amounts of information, such as images or audio files, between the two centers. The main disadvantages of these systems are their dependence on customer communication services and an infrastructure cost higher than other systems.

There are other services in terms of connectivity offered by some companies, such as the service offered by Securitas Direct [15], which detects frequency inhibition systems and immediately transmits the alarm through a network. This high-security alternative is based on a proprietary protocol called the ATN [16] (Alarm Transmission Network), which uses the Sigfox network itself to transmit alarms when sabotage of the main network of the security system is detected. This type of architecture is a system that uses consolidated technologies that allow savings in the initial cost associated with the infrastructure; however, with respect to consumption and the cost of the service, their costs are higher and not very flexible because they are proprietary technologies.

In addition, there are improvements that can be made at one level of the system. On one hand, we have the case of Zigbee [17]. As will be seen in the following sections, the microcontroller selected allows for the use of BLE, Zigbee, and Thread [18], which would allow various technologies depending on the configuration of the building to be controlled. Zigbee would be a suitable option and alternative to use in the development of a WPAN network for mesh network topologies. In the case of a home, a coverage of 10 m will be considered sufficient, so the BLE option with a point-to-point network topology is more feasible, since optimal consumption is achieved and there is more flexibility in sensor hardware. On the other hand, as an alternative technology at the LPWAN level, narrow band Internet of things (NB-IoT) technology is available, which has the advantage of using a strongly consolidated infrastructure. In this case, it will be necessary to use it through the phone carrier network [19], its cost/consumption is like that of Sigfox, with the difference that it is necessary to develop an electronic module adapted to the project.

In the case of Sigfox, we can find a solution based on ESP32 and numerous accessories that allow us to integrate a commercial product without the need for a design stage.

Table 1 shows a comparison of all the systems proposed in this section and the proposed system, the LPWAN–WPAN hybrid network. This table focuses on the details of each of the systems and the most relevant characteristics: energy consumption, transmission rate, coverage, and cost. All of them have strong disadvantages in some of the above aspects. In contrast, our architecture proposal has justified advantages, such as a wide range or an exceptionally low initial cost of service. The transmission rate of the hybrid network is limited by the latest-level gateway developed in Sigfox. However, this aspect is of the least relevance, because the proposed system remains in sleep mode most of the time. The system only wakes up to send a small packet, with enough available bandwidth in Sigfox to keep up with the system needs.

## 3. Architecture

The objective of the proposed architecture is to develop an emergency system capable of detecting fires automatically by measuring parameters such as temperature or gases, at a reduced cost and energy consumption. Therefore, it is necessary to use several types of communication to concentrate the information as much as possible on a single point. In the case of a low-cost emergency system, large coverage is a priority over bandwidth, since traffic is extremely low during active times. Hereby, our proposal focuses on the use of diverse technologies along multiple levels, so that devices can cover a large geographic area and that the cost of services can be divided as more devices are able to be connected to the same system.

The proposed architecture includes a single LPWAN-based Sigfox gateway to manage and listen to tens or hundreds of Bluetooth subnets within a WPAN paradigm formed by measurement nodes and LoRa links. At an economic level, the Sigfox system, as it is a technology with a solid and implanted infrastructure, will allow us to avoid the costs incurred by the implementation of the communication system in the metropolitan area, such as the cost of repetition systems. This will be replaced by an annual service fee for the use of said system (its cost in 2022 was €18.14 including taxes). This cost will not be paid by the user; it will be divided among all homes that are within reach of the last level of architecture. On the other hand, with respect to coverage, LoRa RAW [20] (the LoRaWAN layer is bypassed, the radio is used directly, and the transmitted data are not formatted or encrypted in any way) will allow communication between the WPAN subnets with the Sigfox gateway despite a long distance and obstacles in the environment. The basic steps to build the architecture are detailed next:

1. **Technologies:** Depending on the communication technologies chosen, the location of the devices and their topology may change.
2. **System Structure:** The diverse levels of the communication topology are described.
3. **Hardware:** Once we know the communication technology and topology, we can choose the hardware to accomplish high availability and low energy consumption.

### 3.1. Technologies

The main technologies involved in the communication system proposal are presented next:

WPAN Communication:

A WPAN is a communication technology aimed at transporting data between different devices over a short distance. This technology is designed for communications about meters that do not require a large bandwidth. The most relevant WPAN technology is Bluetooth, which is a standard based on IEEE 802.15.1. Bluetooth is a technology in continuous evolution that allows for transmission rates of the order of Mbps at an incredibly low energy cost, making it the most commonly used WPAN technology today. Regarding the Bluetooth 4.X extension, the use of BLE is available, which is adapted for new communication architectures oriented to the IoT. Table 2 presents the main characteristics of the Bluetooth standard.

In this design, the use of bluetooth low energy (BLE) endows the node with exceptionally low energy consumption that allows devices to be placed in unattended locations and operate autonomously without the need to be charged for extended periods of time. In addition, at present, the market for embedded microcontrollers oriented to wireless communications includes BLE modules, which represents economic savings. At the communication level, this communication protocol, which has great flexibility, already allows the use of mesh configurations that are especially useful when it comes to communicating several devices over long distances despite their limitations, because of the use of pairs as repeaters. In this architecture, if the equipment is connected to a constant power source and there is no dependence on energy storage sources or batteries, the mesh topology can be used. Finally, a star topology was chosen to maximally reduce the energy consumption of the nodes. To avoid coverage problems, a study will be necessary to place all network devices in the most optimal way possible.

LPWAN Communication:

A LPWAN is a set of wireless network technologies that allow the communication of insignificant amounts of data over long distances with extremely low energy consumption. To achieve this, on many occasions, the transmissions are limited to small periods of time with subsequent reception windows.

The relevant technologies in the development of architecture are Sigfox and LoRa, which are briefly described next:

Sigfox is a proprietary LPWAN technology that has an infrastructure already consolidated throughout Europe and mainly in high populated areas. This infrastructure consists of a network of base stations and dedicated servers to receive data. Communication is bidirectional, although asymmetric, since the reception capacity of the devices is less than the transmission capacity on the ultra narrowband (UNB) physical layer.

Sigfox covers a total of 75 countries around the world [21]. Using Sigfox as a link to the cloud allows great savings in the initial cost of implementing the system. In addition, it has great coverage, allowing for the connection of isolated geographical situations. Although its transfer rate is incredibly low, it is sufficient to meet the needs of an emergency system, that is, to transmit emergency situations. Sigfox has reduced energy consumption, which also allows for the possibility of being powered by systems based on energy harvesting or, in the event of a power failure, continuing to transmit due to an auxiliary energy system such as a lithium battery. Table 3 shows the main characteristics of Sigfox technology.

LoRa is a technology that uses unlicensed ISM bands. It uses a modulation patented by Semtech called Chirp Spread Spectrum (CSS), which allows it to be more robust against interference or noise. The transmitter modifies the frequency at which it emits and that allows the receivers to decode the received signals despite their attenuation, so each frequency corresponds to a different distance and transmission speed. Table 4 shows the main characteristics of the LoRa technology.

LoRa technology serves as a bridge between the WPAN subnet and the Sigfox network. Because of its great coverage, it will be able to avoid obstacles and correctly transmit an emergency. The cost of this type of technology, although it is higher than in the case of BLE, is not excessive. In opposition, LoRa consumption is continuous due to its permanent listening state with the nodes, so LoRa nodes must be plugged into an electric source. Regarding its topology, it has great flexibility, as in the case of BLE, providing robustness against system failures. If the LoRa gateway cannot communicate with the Sigfox gateway assigned to its network. It could switch to another gateway of the same communication layer, but of another building. That gateway is assigned to another network, but of the same service, to try to communicate the emergency, thus increasing the robustness of the system.

### 3.2. System Structure

**Figure 2:**
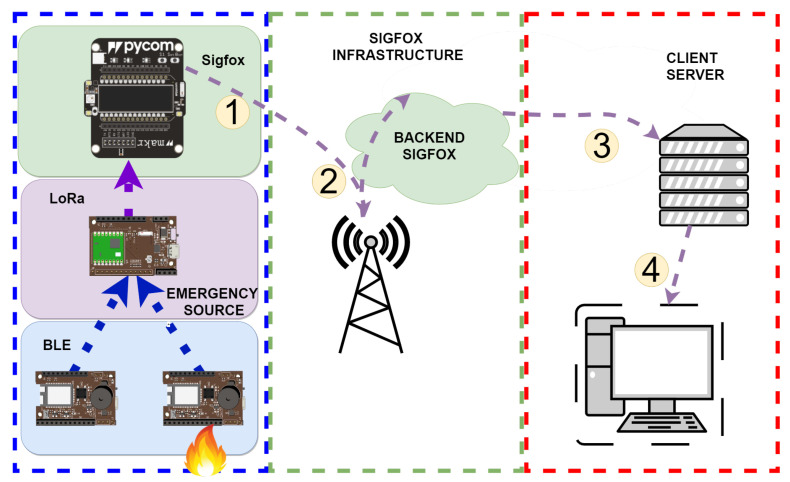
Summary diagram of the proposed emergency system.

The proposed emergency system architecture can be divided into three layers at the device and communication levels. Each of these layers communicates with the next layer using a different WPAN or LPWAN technology. Figure 2 shows a global diagram and all components involved in communication from the emergency point to the destination. In Step 1, communication occurs through the global structure of the physical architecture. In Step 2, the alarm is transmitted through the structure of the service provided by Sigfox. In Step 3, the alarm is processed in the client’s alarm processing and storage structure. Finally, in Step 4, the alarm is displayed at the destination warning system in the emergency center.

#### 3.2.1. Layer 1: WPAN Subnet of Sensor Nodes

**Figure 3:**
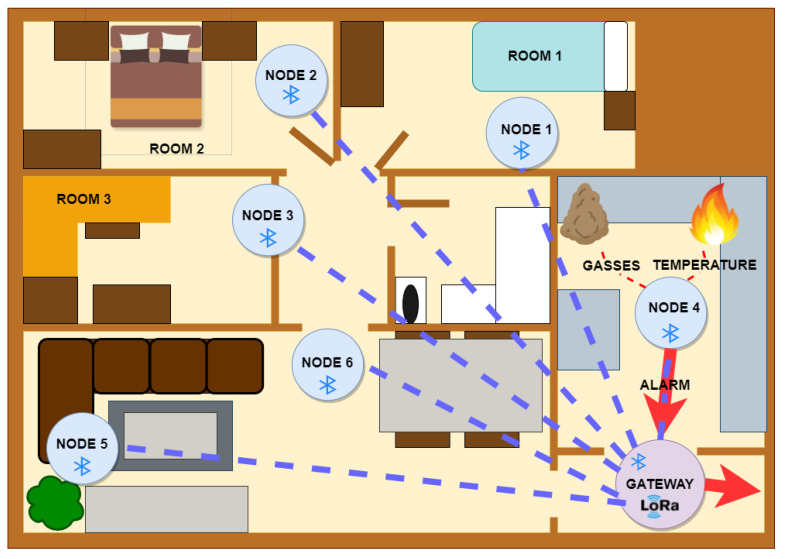
Layer 1: BLE network, star topology.

The first layer of the architecture is composed of a set of node/sensor devices with Bluetooth connectivity and star topology, as shown in Figure 3. These devices collect measurements of the environment, and they also have activator modules made up of push buttons to trigger emergency notifications. The main measure for detecting fires is temperature, smoke, or concentrations of compounds produced in the combustion of materials. If the temperature or any additional measurement exceeds a given threshold, the alarm is activated, and the node sends a message via Bluetooth to the WPAN network gateway.

Regarding the role of each device within the WPAN network, the sensor node device acts as a slave with server functionality; that is, this node is advertised on the network and will wait for the connection request made by the master device. When it detects an alarm, it stores the data and provides access methods to the client. The gateway of the WPAN network acts as a master/center with the client functionality. It will supervise the scanning of the advertised devices within its coverage and will make connection requests to the devices if the service identifier or UUID (universally unique identifier) of the nodes matches the service identifier assigned to the detection devices of the emergency system. Therefore, uniquely, the devices involved can interact with the customer. To give more security to the home network, each WPAN subnet will have a unique UUID. In this way, each existing WPAN network in each home has its own service identifier to avoid connection to nearby devices located in another home. When the gateway receives any alarm data from any of the nodes belonging to its network, the gateway will send a frame over LoRa RAW to notify the last communication layer of the system.

Since the gateway is a device that constantly scans emergency service devices, it should be continuously plugged into a wall power outlet. It is also advisable to incorporate a battery system as backup to avoid power shortages. Figure 3 shows a fire set up in the kitchen and the system detecting the fire. This detection is given by a reduction in air quality or an increase in temperature. The node is advertised to the client / gateway and a BLE connection is established. Through this connection, the alarm is notified to the next layer of the system.

#### 3.2.2. Layer 2: LPWAN LoRa RAW Network in a Star Topology

**Figure 4:**
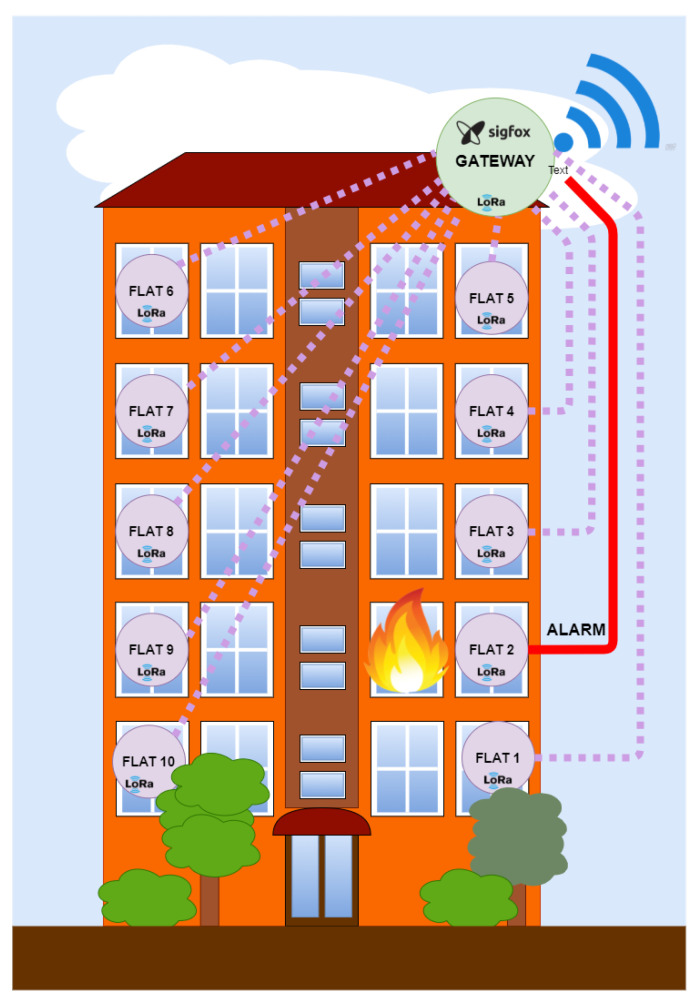
Layer 2: Star LoRa RAW Network.

The second layer is shown in Figure 4. It is formed by the network of devices with LoRa connectivity in a star topology. Each home has a gateway that acts as a BLE client, receiving the information from each server on the existing WPAN subnet. These devices are connected to another gateway that will act as a LoRa client, giving access to the cloud through Sigfox. The reason behind using LoRa in this layer is due to its range despite the existing obstacles in a building, allowing the concentration of all the information from a housing community to a single device at a great distance. The use of WPAN technology would be unfeasible due to the dramatic reduction in coverage due to the presence of obstacles such as walls or floors of the building.

The data frame sent by the LoRa gateway, being a private network, is described below. In addition, LoRa RAW is used as the physical layer of communication. The link layer of the protocol stack is used instead of the network layer. Therefore, this architecture may be considered a private LoRa network in which the resources of the LoRaWAN infrastructure are not used. Due to this, it is necessary to develop a simple communication protocol in which each alarm data frame will be accompanied by information regarding the identification of the network. This fact allows the gateways to differentiate the data received by diverse emergency networks. The assignment of information to the data frame is represented in bytes.

- **IDSERVICE (Byte** 〈**3: 0**〉**):** This represents the header that identifies the service. Messages that do not have the value assigned to the network service will be discarded.
- **IDNETWORK (Byte** 〈**5: 4**〉**):** This identifies the network of each available system. Each set of homes connected to a Sigfox gateway sends the information with this data. It is simply informative since there could be the possibility that a gateway of another system does not work, and these systems can also transmit through another, which provides greater robustness to the system, despite creating duplication in the data.
- **IDGATEWAY (Byte** 〈**6**〉**):** This identifies the gateway that sends the message.
- **IDNODE (Byte** 〈**7**〉**):** This identifies the node that sends the message.
- **CNTMSG (Byte** 〈**8**〉**):** This is a message counter.
- **ALARM (Byte** 〈**9**〉**):** This is a flag that indicates the alarms that have occurred. 0x01: Temperature alarm; 0x02: Gas alarm; 0x04: Battery alarm; 0x08: Push button alarm.
- **CHECKSUM (Byte** 〈**10**〉**):** This ensures the correct transmission of the frame. As the last piece of data, the 2’s complement of the sum of all the previous values of the communication frame is transmitted. This value will be checked at the destination to ensure that the received frame is correct.

This encapsulation is preliminary. In order to increase its security, it could have data encryption.

#### 3.2.3. Layer 3: Access to the Cloud by LPWAN Sigfox

**Figure 5:**
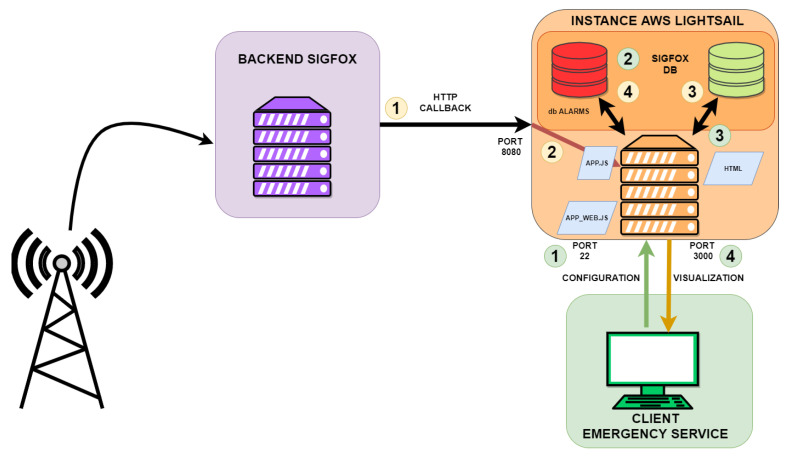
Layer 3: Backend Sigfox and emergency server.

The last layer of the system is formed by a device with dual LPWAN connectivity. The new gateway acts as a LoRa RAW server and a Sigfox client. This device is formed by the commercial board model Lopy4 [24] designed by the company Pycom. It is an ESP32 microcontroller with the possibility of connectivity through BLE, LoRa, and Sigfox. Therefore, this device is ideal for the hybrid system as a gateway between the two LPWAN technologies, receiving LoRa frames, converting them, and sending the suitable information to the Sigfox network. The normal operating mode continuously listens for incoming transmissions from the lower layer of the system. In this mode, the gateway works as a LoRa receiver in Mode C. When any transmission is received, its integrity is verified with a checksum byte, and the notification will be retransmitted to the cloud. Because Sigfox and LoRa transceivers share the same antenna in the Sigfox gateway, it is necessary to change the firmware state machine state from the LoRa receiver to the Sigfox transmitter. This could be a problem in communication systems with continuous transmissions because information blocks could be lost, but in this case, it does not happen since it is a system that is normally at rest and when several messages are sent, they are repeated several times to ensure the arrival of the alarm at its destination. The transmission is sent through the proprietary network from the distributed stations to their servers, where all the transmitted information is correlated with the ID of the registered Sigfox device. At this point, there are several data processing options, which allows for the redirection of information received to a server that owns the service, where a data crossing can be made to know exactly the geographical position or address where the alarm is generated. In Figure 5, the final data transmission process is shown. The alarm is emitted from the affected building to the Sigfox proprietary network, whose destination is the backend of Sigfox. There, a series of rules are consulted to determine how the information received should be treated. In this case, it is relayed to the client server. The information will be stored on this server and the data will be crossed to display the alarm with the exact address where the event has occurred. The yellow bubbles represent the sequence to store Sigfox backend information on the server. On the other hand, the green bubbles represent the string to read the server information from the web client.

Regarding the security of this technology, it has different security methods such as Anti-Replay or Anti-Eavesdropping [25], and each Sigfox device has a unique identifier. On the other hand, connecting this system through the Sigfox network yields a natural firewall because our data is not connected directly to the Internet, providing greater security to the network data.

### 3.3. Hardware

According to the previous subsection, the architecture consists of three layers, and each of them have a different type of device. For the upper layer (Layer 3) and due to the wireless communication technology used, it is convenient to use commercial solutions such as those proposed by Pycom [26] with its lopy4 electronic model, a device that allows communication in various communication protocols in WPANs and LPWANs, including the Sigfox protocol. This device is suitable for the implementation of the last layer of the architecture, since both its hardware and programming libraries allow for the use of LoRa and Sigfox. With this gateway, it will be possible to listen to the lower layers of the LoRa frames to transmit the information to the Sigfox cloud with a single transceiver.

Regarding the hardware electronic parts, instead of using a commercial hardware system, devices in charge of measuring the parameters and communication in the BLE (Layer 1) and LoRa (Layer 2) layers have been designed. This is possible because both the BLE and the LoRa protocols have several built-in options that can be implemented in their control chips.

Given this design, two key points are presented for the design of a system that works for both layers of communication. On the one hand, it requires that the nodes/sensors of the system are energy-efficient, since they can be located at any point in the home or building and therefore cannot receive external or wired power of any kind, thus requiring great autonomy without external power sources (e.g., a lithium battery). On the other hand, the LoRa gateway does not present this problem since it must only be within the BLE subnet coverage area; therefore, this device can be connected at any point of the electrical network.

**Figure 6:**
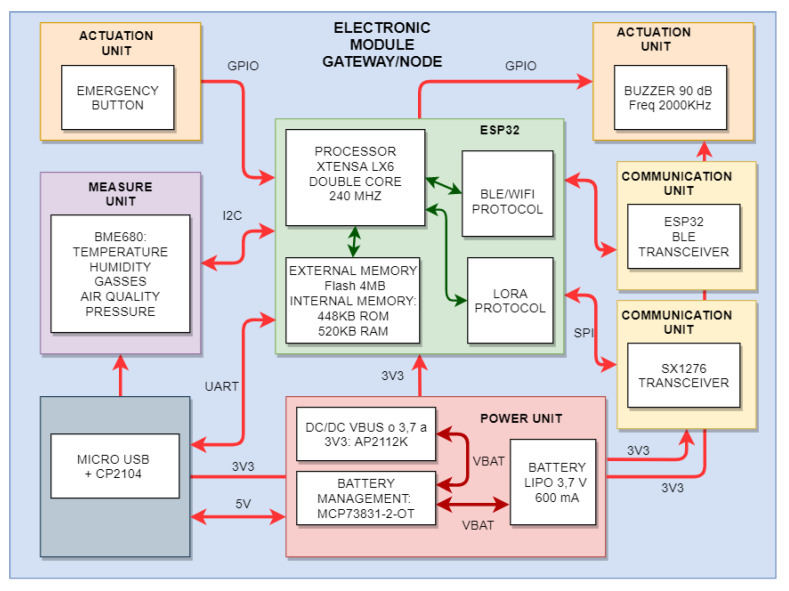
Diagram of node/gateway electronics based on ESP32.

Figure 6 shows the diagram block proposed for the node/gateway hardware prototype. Each block or set is responsible for each of the main functions that a node must contain. These main functions are the power supply, the sensor or measurement unit, the processing unit made by the ESP32 microcontroller, the communication system, made by the transceiver included in the processing unit, and an external transceiver for communication by LoRa when the device works as a gateway. An actuation block is also included, consisting of an emergency button that activates an alarm and a buzzer that notifies the user upon detection of an emergency.

**Figure 7:**
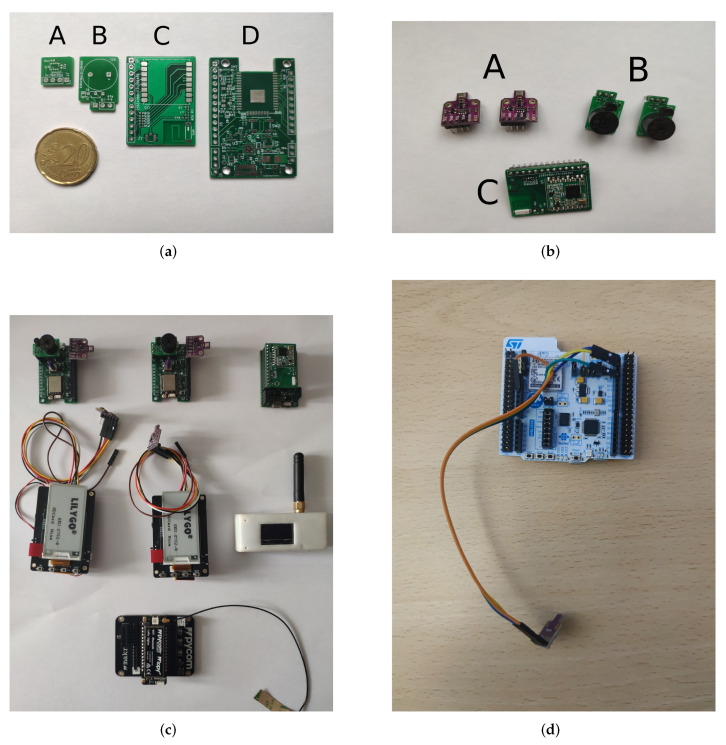
Prototypes based on ESP32 and STM32 microcontrollers. (**a**) Power and microcontroller. A. Temperature Sensor; B. Buzzer; C. LoRa transceiver; D. Main Board. (**b**) Peripherals assembled. (**c**) Devices used for system analysis. (**d**) New node based on STM32 microcontroller.

**Figure 8:**
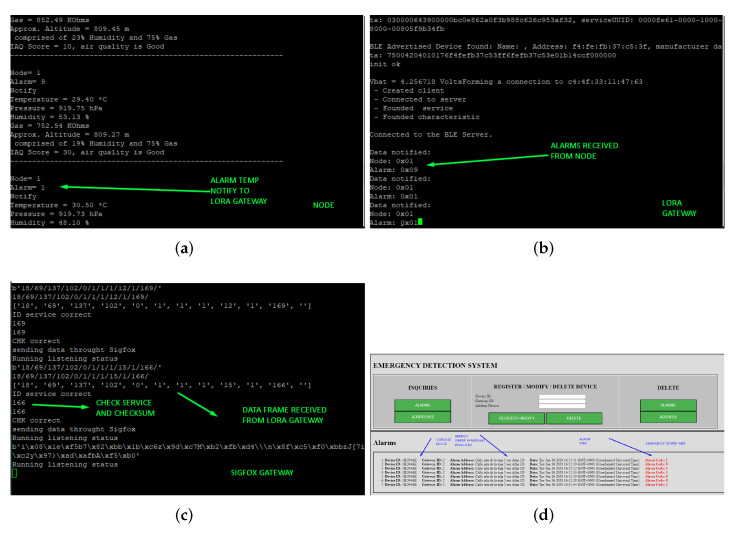
Process of the firmware working on diverse devices of the system. (**a**) Node/sensor idle status. (**b**) LoRa gateway receiving an alarm from the BLE subnet. (**c**) Sigfox gateway receiving a dataframe through LoRa RAW. (**d**) Emergency center/client website.

Therefore, a double solution is proposed to cover the key points of the system:

- At the hardware level, it is necessary to find a microcontroller capable of reducing its average energy consumption to values in the order of µHours in standby mode. The first tests of the system were carried out with an Espressif microcontroller [27], the ESP32 model [28]. This microcontroller has a wide range of peripherals, such as a Bluetooth transceiver. This component is widely used, and throughout the Internet, many software resources for its programming can be found, owing to the community behind this product. The proposed solution allows for the development of both a node and a prototype LoRa gateway (see Figure 7). After the first tests of the system, we detected that its consumption levels, even in its different low consumption modes, were too high for this type of application. Therefore, we decided to use a more efficient alternative. This alternative is a microcontroller model that meets the requirements at the level of connectivity and consumption: the STM32WB55 [29] from STMicroelectronics [30]. This microcontroller has several low-power methods, improving battery life over the previous version to approximately 2300% (see Table 5). For the first tests, an evaluation board was chosen that mounts the model BME680 [31] from the manufacturer Bosch (c.f. Figure 7a,b, PCB A, from Figure 7). Figure 7 shows the prototypes developed. In Figure 7a, the designed PCBs are displayed: the BME680 measurement sensor (**A**), the Buzzer peripheral board for audible alarms (**B**), the LoRa transceiver accessory board (**C**), and the main board that will supervise the processing and powering of peripheral boards (**D**). In Figure 7b, the peripheral boards used are assembled. In Figure 7c, the devices used for the first tests of the system are shown, from purpose-built nodes/gateways to the Sigfox gateway purchased from Pycom. Figure 7d shows the node/sensor prototype based on STM32 whose consumption is much more appropriate according to the energy requirements of the system.

- At the firmware level, the system must switch between different operating modes for optimal energy efficiency. As it is a temperature-based fire detection system, it must wake up every short period of time, in this case, every 30 s, and act in the event of a detecting temperature increment. The system continuously measures the environment to detect any temperature exceeding a set threshold (e.g., 70 °C [32]). If so, the system enables the WPAN transceiver and connects to the LoRa gateway to transmit the alarm. The LoRa gateway always works in active mode as a BLE client, when the server node wants to transmit an alarm, it will be announced so that the gateway client can connect and receive the alarm information. Next, it will generate a frame that will be transmitted by LoRa to the last gateway, where it will be verified that the data are correct. Figure 8 shows an example of alarm transmission, from the node to the web client of the emergency center. Figure 8a shows how the node collects values such as temperature, humidity, or air quality, amongst others. For instance, when a combination of critical parameters reaches a threshold, an alarm occurs and must be notified. According to the generated alarm, a code associated with the type of alarm is sent together with the identifier of the node. Figure 8b shows how a connection with the advertised device is established. The gateway receives two data reported by the node: the identifier and the type of alarm. Figure 8c shows how a byte frame is received; these are the packets previously formed by the LoRa gateway associated with the emergency site. It contains all the information necessary to locate the exact location of the event. This image also shows how a check was previously made where the frame corresponds to the service and that the received data is correct on account of the checksum. Figure 8d displays an example of a client web application that can view the information collected by the system. Due to a cross-over in its databases, the information provided allows for a geographic visualization of the emergency, as well as the emergency code, allowing the emergency service to act according to the generated alarm.

## 4. Results

This paper presents an emergency system based on a hybrid wireless communication system that is robust and benefits from a reduced cost, a long coverage, and low consumption. Thus, an analysis of the most relevant specifications, such as consumption, coverage, latency (response time of the system), and cost, has been conducted. Section 4.1, Section 4.2, Section 4.3 and Section 4.4 present the discussion for each of these specifications, respectively.

### 4.1. Consumption

**Figure 9:**
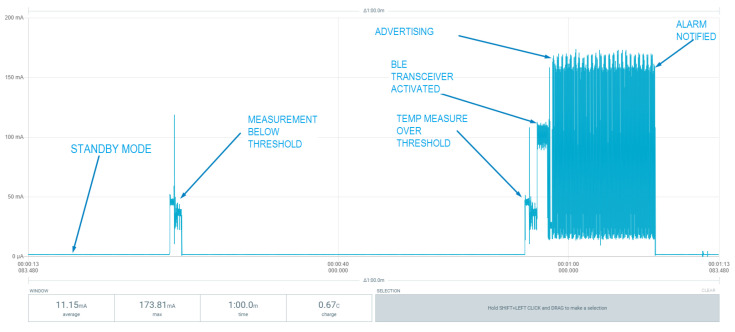
Full communication alarm process.

**Figure 10:**
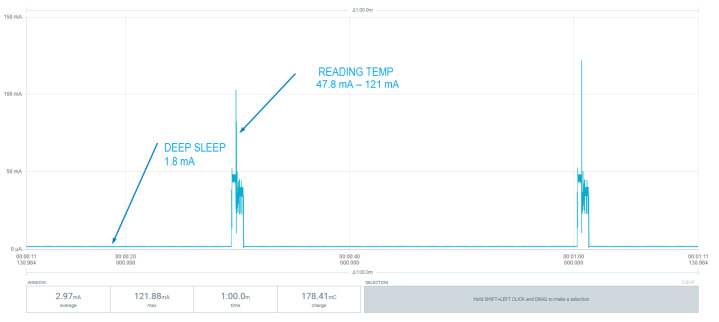
ESP32 node: Low-power mode (Deep Sleep) and sensor reading.

**Figure 11:**
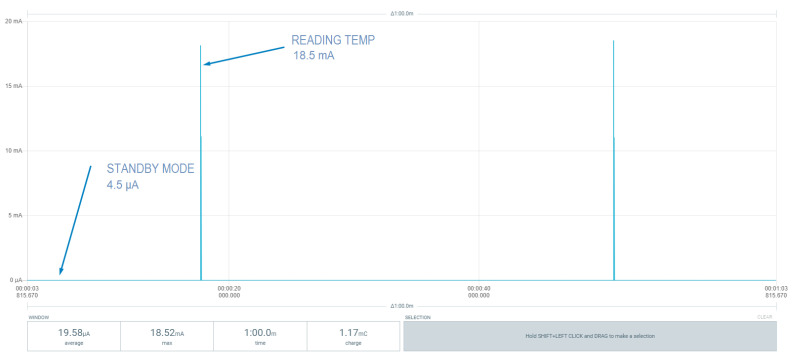
STM32 node: Low-power mode (standby mode) and sensor reading.

Consumption is one of the critical parameters for a node in a wireless network, so its autonomy and energy efficiency must be optimal. In this study, tests have been carried out with two types of nodes: an ESP32-based microcontroller and the microcontroller STM32WB55 of the STM32 family. Both devices must be able to enter into a low-power state where, every 30 s, a reading from the temperature sensor is performed. If the value of that sensor does not exceed the set threshold, it returns to the idle state. Otherwise, it must activate its wireless communication block through BLE to communicate the alarm to the other layers of the system. Figure 9 shows the entire communication process of an alarm by the node through Bluetooth with the ESP32 microcontroller. The alarm communication process consumes a large amount of energy, but it is not in its regular state. The equipment usually works both in low energy consumption or in active mode long enough to perform measurements on the sensor. Figure 10 and Figure 11 present a comparison between the two node versions. Even though the values in Table 5 are not fulfilled, the consumption by the STM32 microcontroller is much smaller than with ESP32. Therefore, the nodes of the emergency system should be based on this architecture. According to the results measured with the STM32-based system, an average consumption of 19.58 µA was obtained during the temperature reading period. With a 3 V, 225 mAh lithium battery, we can achieve approximately 479 days of autonomy, while the battery life is reduced to only 3 days when using the ESP32-based board.

Table 6 shows a comparison of the results obtained with the theoretical consumption extracted directly from the components’ datasheets. A big difference in power consumption can be seen. This difference in consumption is mainly due to two phenomena:

1. The value in standby mode may be higher due to the different leakage currents or consumption of the pull-up resistors that the circuit contains (i.e., the pull-ups of the I2C communication).
2. The difference in value between the consumption at the time of reading may also be due to consumption peaks that occur when the microcontroller wakes up, which might make it necessary to carry out the measurement with higher-resolution instrumentation in order to extend the data acquisition in the temperature measurement period and to read and calculate the value after the current peak during startup.

**Table 6:** Comparison of theoretical and measured power consumption values on the node/sensor (STM32 + BME680).

	STM32 (Standby Mode) + BME680 (Standby)	STM32 (Run1 Mode) + BME680 (Measuring Temp)
Theorical Consumption (mA)	0.00239	7.838
Measured Consumption (mA)	0.0045	18.5

Figure 9, Figure 10 and Figure 11 show the consumption results obtained with the different sensor prototypes used to measure the temperature parameter. Figure 9 plots the entire measurement process: the activation of transceivers, advertising and sending of alarm to the gateway, and the consumption of each process. This entire process was performed with the ESP32-based prototype.

Figure 10 and Figure 11 present the comparison of consumption at the most critical step of the node: the measurement of temperature and sleep state. Both states will be the processes in which the node will normally be and, therefore, will most need to be monitored when predicting the autonomy of the system.

In Figure 11, the node based on STM32 performs the measurements with lower power usage compared to the node based on ESP32 shown in Figure 10. Sleep mode consumption is also lower, allowing the system to achieve an average consumption of µA rather than mA, which ultimately results in greater autonomy.

Table 7 provides a comparison of the nodes developed for this system with a conventional detector. We can see the improvement in energy consumption achieved in current and power.

As observed in Table 7, the ESP32 node and the traditional detector increase their power consumption by a factor of 166 and 1989, respectively, with respect to the STM32 node.

### 4.2. Coverage

In terms of coverage, a key point in the architecture development is the maximum distance capable of reaching each of the subnets of the system. The lower-level network is the accumulation of devices connected through a WPAN network with bluetooth low energy connectivity. In a star topology as the proposed in this work, the maximum range is only few meters, which will be considered enough in most systems for the formation of a WPAN subnet at any address. Nevertheless, in certain homes, due to their size, it will be necessary to use network configurations with a mesh topology that will allow any device on the network to function as a repeater, so the network’s operating area can be increased.

At the next level, the network is configured by an LPWAN network based on LoRa RAW. This level is configured in a star topology since the gateways of the building located in each house are connected to a single Sigfox gateway. The coverage of LoRa devices in the open field is very wide, but when they are indoors, their signal is attenuated by the natural obstacles of the building. Therefore, when LoRa devices are installed, an assessment of attenuation needs to be performed in order to get enough signal level in the devices to achieve the desired performance in the system. Ideally, the installation of these devices is located near windows, terraces, or courtyards to avoid the attenuation of the LoRa signal. For instance, in tests carried out with a transmission power of 14 dBm and an antenna with a gain of 2 dBi, a successful transmission between gateways was achieved, bypassing five floors of a building.

Finally, the last level of architecture, its outdoor coverage, is very wide, reaching tens of kilometres. The Sigfox device is located on the roof of buildings, and the coverage of the proprietary infrastructure is consolidated in Europe. The proposed system with an outdoor-adapted antenna will be sufficient to be able to correctly transmit the generated alarms.

### 4.3. Latency

In this application, the measurements focus on temperature variations. A 35-degree threshold has been set to ease the reproduction of the alarm. Other parameters can cause an alarm, such as the push button installed in the node used to indicate an emergency. Furthermore, the BME680 sensor is capable of measuring the concentration of carbon dioxide, its value can be modified by exhaling breath close to it. For a commercial product, these thresholds must be studied. In the case of alarm detection by a temperature threshold, it could be programmed at 57 °C [33]. With these alarm simulation possibilities and with thresholds set to easily reproduce the alarm, several tests were carried out simulating various types of emergencies. We conclude that the transmission time of the alarm needed to reach the emergency service, starting from the detection of the node emergency, is less than 5 s on average. These values coincide with measurements taken in Saavedra’s research on the different characteristic parameters of the communication technologies [34]. Nevertheless, the transmission time is subject to several factors, such as the situation of the system with respect to the antennas of the proprietary infrastructure or the saturation of the network, but even so it can be concluded that, because of its fast transmission, the response time by the emergency services will be shorter than traditional systems, in which the human factor is involved in communicating the alarm.

### 4.4. Cost

In terms of cost, we can make the following classification:

1. Device Cost: Node/sensor and BLE/LoRa Gateway: Both devices can be considered the same hardware because their core is the same; however, depending on the function, this core is complemented by a peripheral. If the core is used as a sensor node, Buzzer, button, and the BME680 sensor are connected. If it is used as a gateway, only the LoRa transceiver is connected. The cost [35] of these prototypes is as follows:
  (a) Node/sensor: €40.56
  (b) Gateway: €37.41. This price includes: PCB, components, assembly, and taxes; however, this price will change if it is manufactured as a product, since the price of the components is considerably reduced if purchased in larger quantities.
  (c) LoRa/Sigfox Gateway: This device is composed of the following:
    i. Pycom Lopy4: €34.95
    ii. Pymark v3: €16
    iii. Battery: €5
    iv. Waterproof case: €22
  The total price is: €77.95. This price includes a 1 year subscription to the Sigfox service.
2. Service/proprietary infrastructure cost: Mainly two services are needed:
  (a) Subscription to Sigfox: €18.14 including taxes. This service is necessary to use the Sigfox network and services for one year.
  (b) AWS Linux server: The system needs a server with a fixed IP to receive the notifications, storage, and display of the alarms to the end user. Its cost is approximately 4€ per month (total price: €66.14 year).

## 5. Conclusions and Future Work

This paper presents an emergency system proposal that uses a hybrid communication system made up of different WPAN and LPWAN technologies, providing significant advantages due to the low amount of information sent. The main contribution of this work includes a low-cost, energy-efficient, reliable, and high-coverage system.

Bluetooth low energy technology continues to evolve, achieving lower costs and better consumption than its predecessors. Its flexibility enables the creation of different types of networks, allowing the nodes to work as repeaters. This development has allowed us to create architecture at a low cost. At the hardware level, the costs can be quantified in tens of euros for the infrastructure needed in each home. The cost of the service does not depend on other existing services in the house such as Wi-Fi, and it is only necessary to pay €18.14/year for each Sigfox gateway, which can manage the alarms of an entire building. Hence, this annual cost can be shared with multiple houses. Regarding electricity consumption, the second node/sensor has been designed with great autonomy, which allows operation for more than a year without maintenance if no alarm occurs.

According to the obtained results, different design parameters have been analyzed: consumption, coverage, latency, and cost. For consumption, different tests were carried out with two types of nodes, taking into account their real use and important information about their autonomy. A coverage analysis was performed, taking into account outdoor and indoor use and assessing the proposed architecture. As far as latency is concerned, the analysis shows that a fast transmission produces a shorter response time of emergency services than traditional systems, in which the human factor is involved in alarm communication. Regarding cost, both device and service/infrastructure costs have been analyzed, taking into account the proposal.

Despite having achieved a fully functional system, due to its potential, this proposal is only the beginning of a path for a global system that can improve or increase its functions throughout its implementation or subsequent useful life. In future work, we plan, among other tasks, to do the following:

- implement LoRaWAN as a final technology as a unique and global service;
- upgrade the architecture with bidirectional communication to check system status, configuration setup, and request relevant information;
- build an energy harvesting system to increase the devices’ autonomy using renewable sources such as solar panels or wind turbines;
- integrate additional environmental measurement sensors (e.g., gas, passive infrared, ultrasound, and medical sensors).

## Figures and Tables

**Table 1:** Relevant characteristics and comparison between the state of the art and the proposed system. Features: worst (red), poor (orange), medium (yellow), and best (green).

N: Node G: Gateway	Ferreira et al. [8]	Semtech [9]	Securitas Direct [15]	Muheden et al. [10]	Heyitech Alarms [14]	Hybrid Network (Our Proposal)
Telecommunications technology	N: WPAN(BLE) G: LPWAN(LoRa)	LPWAN: LoRa	LPWAN: NB-IoT, Sigfox, LoRa	N: WLAN (Wi-Fi) G: WAN (Fiber)	N: WPAN/ RF owner G: Cellular 2G/3G/4G	N: WPAN(BLE) G1: LoRa G2: Sigfox
Transmission rate	N: 1, 2, 3 Mbps G <50 kbps	<50 kbps	LoRa <50 kbps Sigfox <1 kbps/day NB-IoT <200 kbps	N: 802.11ac <866 Mbps G: 802.11b <1 Gbps	N: <1 Gbps G <10 Mbps	N: 1, 2, 3 Mbps G1 <50 kbps G2 <1 kbps/day
Range	N: 10–100 m G: 5–20 km	5–15 km	N: 10–100 m G: 5–20 km	N: 10–100 m G: 10–40 km	N: 10–100 m G: 5–15 km	N: 10–100 m G: 10–40 km
Energy Consumption	N: very low G: very low	Very Low	Very Low	N: Medium G: High	N: Low-Medium G: High	N: very low G1: very low G2: very low
Cost D: Device S: Service I: Infraestructure	D: Very low S: None I: High	D: Low S: None I: Very High	D: Low S: Sigfox €18.14 gate/year LoRa: None NB-IoT Very High I: Sigfox: None LoRa: High NB-IoT: Low	D: Low S: €30/month/gate I: none	D:Medium S: €50 /month/gate I: €250 + €30 /month/gate	D: Very low S: €18.14/n houses gate/year I: None

**Table 2:** Main characteristics of Bluetooth.

Feature	Value
Working Frequency	2.4 GHz
Coverage	100–200 m (open) 10–20 m (with obstacles)
Transmission rate	Up to 1 Mbps (Bluetooth 4.2) 2 Mbps (Bluetooth 5.0)
Network topology	Point-to-point, star or mesh

**Table 3:** Main characteristics of Sigfox [21,22].

Feature	Description
Communication	Bidirectional through APIs
Working frequency	RC1: 868 MHz–878.6 MHz RC2: 902 MHz–904 MHz RC3: 922 MHz–923.5 MHz RC4: 920 MHz–922 MHz RC5: 922 MHz–923.5 MHz RC6: 865 MHz–867 MHz
Coverage	10 km (Urban)–40 km (rural)
Payload	12 bytes (140 messages per day)
Transmission rate	100 bps.
Reception rate	600 bps.
Channels	400

**Table 4:** Main characteristics of LoRa [22,23].

Feature	Description
Communication	Bidirectional
Working frequency	433 and 868 MHz (Europe), 915 MHz (America)
Coverage	5 km (Urban)–20 km (rural)
Transmission rate	27 kbps
Topology	Mesh Star of stars.
Device types	Class A: Oriented to transmission exclusively. Class B: Oriented to transmissions in time windows Class C: Devices oriented to continuous listening to receive information. It does not have any consumption limitation.
Channels	10 (Europe), 80 (USA) and 9 (China)

**Table 5:** Comparison of power consumption between the proposed microcontrollers.

Operating Mode	ESP32	STM32
Active (Rx/Tx)	80–120 mA	4.5–5.2 mA (2300%)
Active (Without transc)	10–20 mA	2–3 mA (660%)
Sleep (ESP32)/Stop (STM32)	0.8 mA	2.1 µA (40,000%)
Deep Sleep (ESP32)/Standby (STM32)	0.15 mA	600 nA (25,000%)
Shutdown	2.5 µA	13 nA (19,200%)

**Table 7:** Comparison of power consumption between conventional detectors and designs. * Values calculated from official technical specifications.

	STM32 Node/Sensor	ESP32 Node/Sensor	IoT Milesight EM300th Wireless [7]	Traditional Detector 1 (Unipos FD3010) [6]	Traditional Detector 2 (Honeywell Edam 100) [5]	Traditional Detector 3 (Beinat) [4]
Average consumption (mA)	0.0196	2.97	0.091 *	0.040	0.08	9.77 (Idle)
Power consumption (mW)	0.0588	9.801	0.32 *	0.9	1.92	117
